# Commentary: Safe conception in the U=U Era—HIV transmission risk, reproductive intentions and pregnancy planning in Southern Ghana

**DOI:** 10.1177/17449871261420078

**Published:** 2026-04-28

**Authors:** Ngcobo Seluleko Eric

**Affiliations:** Oxford Brookes University, UK; The University of Cape Town, South Africa

**Commentary to:** Safer conception needs of women living with HIV in Southern Ghana: a qualitative study (Klutsey et al., 2026).

The paper ‘Safer conception needs of women living with HIV in Southern Ghana: a qualitative study’ (Klutsey et al., 2026) offers a timely contribution to the literature on reproductive health and HIV in sub-Saharan Africa. Through in-depth interviews with 24 women living with HIV (WLHIV) in Ghana, the authors illuminate how childbearing desires remain a concern for WLHIV in Ghana, despite great advancement in research and HIV interventions that enable WLHIV to carry full term pregnancies without infecting their child. This paper demonstrates the gaps in health systems worsened by the lack of knowledge and information sharing between health practitioners (in this instance, nurses) and WLHIV. In addition, the findings are valuable for nursing audiences because they demonstrate that safer conception is not merely a biomedical intervention, but a relational, communicative, and ethical form of care that needs to be promoted across Ghana.

## Summary of the paper

The study explores the safer conception needs of WLHIV who desired childbearing and to have biological children within the next 2 years from the start of the study. Using an exploratory qualitative design and thematic analysis, the authors identify four interrelated domains of need: (1) confidential and healthcare worker-initiated communication, (2) education on safer conception strategies, (3) couple-based education and disclosure support, and (4) system-level enablers such as empathy, uninterrupted antiretroviral therapy (ART) and assistance with infertility.

Women in the study described strong desires for motherhood, often prioritising childbearing despite awareness of HIV transmission risks. However, similar to other study findings in a neighbouring country, Uganda (Mustapha et al., 2018), most participants lacked accurate knowledge of safer conception strategies such as timed unprotected intercourse, viral suppression, or partner testing. Many reported fear, confusion, and moral distress when attempting pregnancy, frequently resorting to prayer as coping mechanism. Importantly, participants described clinic environments as a rushed routine by judgemental nurses which discouraged them from raising fertility intentions. In some cases, women disengaged from care or defaulted ART while trying to conceive, underscoring health decision-making without information or guidance and support from health care, and how unmet childbearing needs without healthcare guidance undermine HIV prevention goals (Mbonye et al., 2010).

The findings strongly resonate with HIV-related healthcare professionalism that emphasise relational care, such as person-centred and ethics-of-care frameworks (Egan, 1982; Kangasniemi et al., 2015; Lachman, 2012; Ulrich et al., 2010). Nursing practice is shown here not as a neutral delivery of protocols, but as a ‘professional’ practice where the style of communication and moral judgement directly shape health outcomes. Similar findings of nurse’s attitudes directly impacting uptake of healthcare have been recorded to impact healthcare uptake of women elsewhere (Ngcobo and Shumba, 2023). Women’s accounts of being scolded, laughed at, or denied ART refills highlight how dangerously punitive and dismissive interactions contradict the foundational nursing principles of beneficence and non-maleficence (Kangasniemi et al., 2015).

From a regional perspective, these findings closely align with evidence from sub-Saharan African countries such as South Africa, Kenya, and Uganda - where provider discomfort, stigma, and protocol-driven counselling have repeatedly been shown to silence fertility discussions among people living with HIV (Goggin et al., 2014; Kawale et al., 2015; Matthews et al., 2012; Mindry et al., 2017). As in South African studies, routine prevention messaging focused on condoms and abstinence appears to crowd out conversations about reproduction; however, strong communications such as sharing of knowledge that an undetectable viral load equals untransmissible, known as the (U=U) messaging and a wide access to ART have proven positive for WLHIV who desire to engage in childbearing in South Africa and elsewhere (Hui, 2023; Jones et al., 2025; Mustapha et al., 2018; Okamoto et al., 2024; Okoli et al., 2021; Onoya et al., 2023; Siegfried et al., 2011; Waitt et al., 2018).

What is particularly striking in this Ghanaian context is the persistence of misconceptions about serodiscordance and viral suppression, even among women established on, and adhering to, ART. This suggests that biomedical interventions alone do not translate into lived safety without deliberate communication, and in the case of Ghana, a strong nurse-led education and information sharing and ethical practice of care. The study therefore reinforces existing research showing that safer conception knowledge is not passively absorbed but must be actively taught, revisited, and contextualised (Schwartz et al., 2017).

## Who can benefit from this research?

This paper is especially relevant for nurses and midwives working in HIV care, antenatal services, and reproductive health clinics. I suggest a multi-professional approach that will involve grassroot education through active community workers, peer educators and other forums of HIV-education to strengthen knowledge sharing and allow WLHIV to make informed decisions (Kebede et al., 2021). It provides concrete evidence that safer conception should be understood as part of routine nursing care rather than a specialist addition. Nurses are often the primary point of contact for WLHIV, positioning them uniquely to initiate fertility discussions, normalise reproductive aspirations and provide accurate guidance.

For nursing education, the findings underscore the need to integrate safer conception, HIV disclosure counselling, and stigma reduction into pre- and in-service training. Without these competencies, nurses may inadvertently reinforce fear, silence, or disengagement from care. The paper also highlights the importance of communication skills that support confidential, one-on-one discussions, particularly in overcrowded clinics where group-based information sharing dominates.

At the policy level, the study speaks to gaps in the Ghana national HIV strategies that focus heavily on prevention of mother-to-child transmission (PMTCT) while neglecting the crucial conception stage and more importantly adherence to ART. As demonstrated here, women often conceive outside formal PMTCT pathways, making safer conception and counselling crucial before pregnancy. Policymakers can use these findings to justify integrating safer conception into service delivery and addressing punitive approaches to ART adherence that ultimately increase transmission risk; while ensuring that nurses are trained to deliver accurate information tailored to WLHIV that desire childbearing.

The study is highly relevant to WLHIV themselves, particularly those in undisclosed relationships or with HIV negative partners. By documenting women’s voices, the study challenges scientifically unfounded assumptions that fertility among WLHIV is irresponsible and rare. Instead, it reframes childbearing as a legitimate aspiration that demands knowledge sharing, and supportive points of care with skilled, compassionate nursing support.

## Conclusion

This study makes a compelling case that safer conception intersects with proper clinical interventions, education of both WLHIV and service providers in different degrees, ethical nursing care, and a health system designed to capture and support WLHIV that desire childbearing. By centring the lived experiences of WLHIV in Southern Ghana, the authors show how silence, stigma, and system constraints can undermine both reproductive rights and HIV prevention. For nursing practice in sub-Saharan Africa, the message is clear: supporting safer conception for WLHIV is not optional, but essential to high-quality, woman-centred HIV care.
